# The ERRα–VDR axis promotes calcitriol degradation and estrogen signaling in breast cancer cells, while VDR‐CYP24A1‐ERRα overexpression correlates with poor prognosis in patients with basal‐like breast cancer

**DOI:** 10.1002/1878-0261.13013

**Published:** 2021-07-16

**Authors:** Katia Danza, Letizia Porcelli, Simona De Summa, Roberta Di Fonte, Brunella Pilato, Rosanna Lacalamita, Simona Serratì, Amalia Azzariti, Stefania Tommasi

**Affiliations:** ^1^ Molecular Diagnostics and Pharmacogenetics Unit IRCCS Istituto Tumori Giovanni Paolo II Bari Italy; ^2^ Laboratory of Experimental Pharmacology IRCCS Istituto Tumori Giovanni Paolo II Bari Italy; ^3^ Laboratory of Nanotechnology IRCCS Istituto Tumori Giovanni Paolo II Bari Italy

**Keywords:** breast cancer, calcitriol, CYP24A1, ERRα, VDR

## Abstract

Vitamin D is used to reduce cancer risk and improve the outcome of cancer patients, but the vitamin D receptor (VDR; also known as the calcitriol receptor) pathway needs to be functionally intact to ensure the biological effects of circulating calcitriol, the active form of vitamin D. Besides estrogen receptor alpha (ERα), estrogen‐related receptor alpha (ERRα) has also been shown to interfere with the VDR pathway, but its role in the antitumor and transactivation activity of calcitriol is completely unknown in breast cancer (BC). We observed that ERRα functionally supported the proliferation of BC cell lines and acted as a calcitriol‐induced regulator of VDR. As such, ERRα deregulated the calcitriol–VDR transcription by enhancing the expression of CYP24A1 as well as of both ERα and aromatase (CYP19A1) in calcitriol‐treated cells. ERRα knockdown limited the effect of calcitriol by reducing calcitriol‐induced G0/G1 phase cell cycle arrest and by affecting the expression of cyclin D1 and p21/Waf. The interactome analysis suggested that Peroxisome Proliferator‐Activated Receptor Gamma Coactivator 1‐α (PGC‐1α) and Proline‐, glutamic acid‐, and leucine‐rich protein 1 (PELP1) are key players in the genomic actions of the calcitriol–VDR–ERRα axis. Evaluation of patient outcomes in The Cancer Genome Atlas (TCGA) dataset showed the translational significance of the biological effects of the VDR–ERRα axis, highlighting that VDR, CYP24A1, and ERRα overexpression correlates with poor prognosis in basal‐like BC.

## Graphical abstract

Our study investigates the molecular effects elicited by the vitamin D derivative calcitriol in patients with breast cancer (BC). We showed that ERRα deregulated calcitriol/VDR‐dependent transcription, causing estrogen pathway activation and upregulation of the calcitriol degradation enzyme CYP24A1. We identified the ERRα/VDR‐interacting proteins by bioinformatics analysis and further demonstrated that simultaneous overexpression of VDR, CYP24A1, and ERRα correlated with poor prognosis in patients with basal‐like BC.

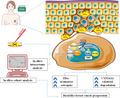

**Abbreviations**
BC: breast cancer
BLBC: basal‐like subtype of BC
BRCA1: breast cancer type 1 susceptibility protein
DMEM: Dulbecco's modified Eagle's medium
ERRα: estrogen‐related receptor alpha
ERα: estrogen receptor alpha
ESR1: ERα gene name
ESRRA: ERRα gene name
LSD1/KDM1A: lysine (K)‐specific demethylase 1A
NC: negative control
PELP1: proline‐, glutamic acid‐, and leucine‐rich protein 1
PGC‐1α: Peroxisome Proliferator‐Activated Receptor Gamma Coactivator 1‐α
PPARGC1A: PGC‐1α gene name
PSICQUIC: Proteomics‐Standard‐Initiative‐Common‐QUery‐InterfaCe
RXR: retinoid X receptor
siRNA: small interfering RNA
TCGA: The Cancer Genome Atlas
VDR: vitamin D receptor

## 1 Introduction

Breast cancer (BC) still remains a deadly disease despite the significant advances in treatment strategies [1]. Hence, the molecular mechanisms of cancer progression need to be further explored and potential biomarkers identified to improve diagnosis and the prognostic classification of breast cancers.

Recently, a large body of epidemiological studies has highlighted a strong association between vitamin D deficiency and increased risk of breast cancer development as well as worse outcome [2, 3]. Therefore, much attention has been directed to using vitamin D to reduce cancer risk and improve the prognosis and outcome of breast cancer patients [4]. In addition to its classic role in regulating mineral homeostasis and bone metabolism, vitamin D is known to exert several antiproliferative and prodifferentiating effects through its derivative, the steroid hormone calcitriol, in a wide range of tumors including breast cancer. The anticancer activity of calcitriol is mostly mediated via genomic actions through binding to the vitamin D receptor (VDR) and activation of the VDR and retinoid X receptor (RXR) heterodimeric complex, which in turn recruits cofactors on vitamin D response elements to induce the expression of target genes [5]. Numerous studies have highlighted that high expression levels of VDR in breast cancer tissues are associated with favorable tumor‐related prognostic factors and a decreased risk of breast cancer death [6, 7, 8, 9]. The antitumor effects of the vitamin D pathway also depend on the levels of the CYP24A1 catalytic enzyme that maintains the levels of circulating calcitriol stable through its conversion to inactive metabolites [10]. Nevertheless, the significance of CYP24A1 expression level as an independent prognostic factor in breast cancer is still a matter of debate [11, 12, 13]. The mechanisms by which the calcitriol/VDR axis promotes protective actions from breast cancer are numerous [14], though interference with estrogen receptor signaling and with aromatase enzyme (CYP19A1) activity [15] has been frequently described. Recent studies have reported that calcitriol can inhibit proliferation of ER‐negative cell lines [16, 17] and have shown that calcitriol induces the expression of functional ERα in such cells, thus suggesting that the growth‐suppressive action of calcitriol is not solely mediated through the ER pathway in breast cancer. Because of its functional kinship with ERα, much attention has been focused over the past decade on ERRα (estrogen‐related receptor alpha) as an important biomarker in ER‐negative breast cancer [18]. ERRα is a constitutively active nuclear receptor, still lacking a natural ligand, which controls the expression of genes involved in oxidative phosphorylation, lipid metabolism, and the tricarboxylic acid cycle. Growing evidence suggests that ERRα plays a central role in coordinating oncometabolic programs that fuel cancer cell proliferation, migration, and metastasis [19], apart from being an important component of proliferative signaling networks [20]. High levels of ERRα expression are associated with a poor prognosis in breast cancer [21], while several reports have described ERRα as a predictive biomarker of response to endocrine therapy in the same setting [22, 23, 24]. Recent studies have described a novel cross talk between ERRα and the vitamin D pathway in diabetes [25]. Astninski *et al*., indeed, demonstrated that the induction of CYP24A1 by fasting was regulated through a (PGC‐1α)‐ERRα‐dependent mechanism, showing, for the first time, a role for ERRα in the suppression of vitamin D signaling. Among interactors of VDR, Battaglia *et al*. [26] highlighted the role of Lysine‐specific demethylase 1A (LSD1/KDM1A), in the corruption of VDR activity in prostate cancer, and Carnesecchi *et al*. [27, 28] reported a close interaction between ERRα and LSD1 to regulate each other, mostly in aggressive cancers. Collectively, these findings prompted us to evaluate the function of ERRα in the deregulation of the VDR signaling network in breast cancer *in vitro* and through a bioinformatics approach to explore the relevant interactions underlying the biological behavior of ERRα. Our findings have demonstrated that ERRα serves the cytotoxic activity of calcitriol while acting as a regulator for VDR to boost the expression of CYP24A1 and trigger that of ERα and aromatase. More importantly, starting from the hypothesis that ERRα overexpression may induce drastic changes in VDR genomic actions, our bioinformatics analysis revealed that simultaneous ERRα/VDR/CYP24A1 overexpression is significantly correlated with shorter survival in patients.

## 2 Materials and methods

### 2.1 Cell cultures

Human breast cancer MCF7 cell line was purchased from ATCC (LGC European partner of ATCC, Milano, Italy, Europe). SUM149PT cells were purchased from Asterand (Detroit, MI, USA). The MDA‐MB‐231 breast cancer cell line and the bona fide normal breast cell line MCF 10A were generously gifted to us by S. Reshkin, Dipartimento di Bioscienze, Biotecnologie e Biofarmaceutica—University of Bari. The cell lines were stored in liquid nitrogen at very early passages before use.

### 2.2 Cell culture conditions

MCF7 were cultured in ATCC‐formulated Eagle's Minimum Essential Medium supplied with 0.01 mg·mL^−1^ human‐recombinant insulin. MDA‐MB‐231 cells were grown in Dulbecco's modified Eagle's medium (DMEM) high glucose, supplemented with NaHCO_3_ (3700 mg·L^−1^) and sodium‐pyruvate (1 mg·mL^−1^). MCF 10A cells were grown in DMEM, high glucose, supplemented with NaHCO (3700 mg·L^−1^), 40 units·mL^−1^ insulin, 0.5 g·mL^−1^ hydrocortisone, 10 ng·mL^−1^ epidermal growth factor, l‐glutamine (2 mm), and sodium‐pyruvate (1 mg·mL^−1^). SUM149PT were grown in Ham's F12 supplemented with 5 µg·mL^−1^ insulin, 1 µg·mL^−1^ hydrocortisone, 10 mm Hepes, and 2 mm l‐glutamine. All medium were supplied with 10% FBS (Gibco, Life Technology, Breda, The Netherlands) and 1% penicillin/streptomycin and then cells were cultured at 37 °C in humidified air with 5% CO_2_ and routinely tested for mycoplasma contamination.

### 2.3 Treatments

Calcitriol was purchased from Selleckchem (Munich, Germany). Stock solutions were prepared in DMSO and were stored at −20 °C until use. For the clonogenic survival assays and IC_50_ value determinations, the MCF7 cell line was treated with calcitriol at a dose range of 0.001–100 nm and the SUM149PT cell line at a dose range of 0.001–1000 nm. Both cell lines were maintained in culture for 2 weeks and treated every 3 days. calcusyn software (BIOSOFT, Cambridge, GB, UK) was used to determine the concentration value yielding 50% inhibition of cell clonality (IC_50_). All the cell lines were treated with 100 nm of calcitriol/vehicle (DMSO) for 4 h/24 h for the gene expression assays. One hundred nanomolar of calcitriol/vehicle (DMSO) was used for 24 h for the cell cycle analyses and cell target modulations. One hundred nanomolar calcitriol/vehicle (DMSO) was used for 1/4 h for the immunofluorescent detection of ERRα and VDR. For the evaluation of sensitivity to calcitriol in transfected cells, IC_50_ concentrations of calcitriol were added every 3 days to the wells in which the transfected cells [negative control (NC) and targeting] had been seeded.

### 2.4 Six‐well colony formation assay

To evaluate the colony formation after treatment with calcitriol, 250 breast cancer cells were plated into six‐well plates, allowed to attach overnight and then treated with scalar concentrations of calcitriol every 3 days, and cultured in a humidified atmosphere containing 5% CO_2_ at 37 °C for 2 weeks. The effect of ERRα silencing on cell clonality and calcitriol sensitivity was tested by seeding 500 cells that had been transfected with small interfering RNA (siRNA)‐targeting ERRα or with an empty vector (NC) and then treated or not with calcitriol at IC_50_ concentrations. After the treatments, the colonies were washed twice with PBS and then fixed in 100% ethanol and stained with crystal violet 0.2%. Visible colonies were then counted. Triplicate wells were counted for each treatment group, and the number recorded was subjected to statistical analysis.

### 2.5 RNA extraction and quantitative real‐time PCR

Total RNA was isolated from breast cancer cell lines using the RNeasy Plus Mini kit according to the manufacturer's protocol (Qiagen, Hilden, Germany). Concentrations were estimated with the ND8000 Spectrophotometer (NanoDrop Technologies, Thermo Fisher Scientific, Waltham, MA, USA). For the transcript‐level analyses, 500 ng of total RNA were reverse transcribed using the High Capacity cDNA Reverse Transcription Kit according to the manufacturer's protocol (Thermo Fisher Scientific, Waltham, MA, USA). Quantitative real‐time PCR was performed on the ABI Prism 7000 Sequence Detection System in accordance with the manufacturer's instructions (Thermo Fisher Scientific). The qPCR assay IDs used were the following: human CYP24A1 (Hs00167999_m1), human VDR (Hs00172113_m1), human RXRA (Hs01067640_m1), human ESR1 (Hs00174860_m1), human ESRRA (Hs01067166_g1), human CYP19A1 (Hs00903411_m1), and human KDM1A (Hs01002741_m1). RN18S1 (Hs03928985_g1) was used as the endogenous reference. Gene expression levels were quantified by the comparative δδCt method after normalization for the endogenous reference. All the PCRs were performed in duplicate for three times. The experiments with calcitriol were performed by using the untreated cells as control, the cells treated with vehicle (DMSO), and calcitriol‐treated cells, and the analysis of data was performed by the δδCt method. Basically, any ΔCt was normalized with the housekeeping Ct (i.e., ΔCt = Ct_target_ − Ct_housekeeping_), instead δδCt for any analysis was calculated according to the following formulas:

$$\Delta {\text{Ct}}_{\text{vehicle}}‐\Delta {\text{Ct}}_{\text{untreated}},$$

$$\Delta {\text{Ct}}_{\text{calcitriol}}‐\Delta {\text{Ct}}_{\text{untreated}}.$$

### 2.6 Immunofluorescence

Experiments were performed essentially as described in Porcelli *et al*. [29]. The MCF7 and SUM149PT cells were seeded onto glass Lab‐Tek Chamber Slides (8 wells; 0.8 cm^2^/well) at a density of 20 × 10^4^ cells per well and incubated for 1 to 2 days at 37 °C. After treatment, the cells were washed twice with HBSS solution and fixed with 3.7% formaldehyde in PBS for 15 min at room temperature. The cells were then permeabilized with Triton X‐100 [0.1% (w/v)] in PBS for 5 min at room temperature. Nonspecific binding sites were blocked for 30 min at room temperature with PBS containing 5% bovine serum albumin (BSA), and the cells were then incubated with a rabbit anti‐VDR monoclonal antibody and mouse anti‐ERRα monoclonal antibody in PBS containing 4% BSA for 60 min at room temperature. VDR and ERRα immunostaining was followed by incubation with Alexa Fluor 488 goat anti‐rabbit antibody (Invitrogen, Eugene, Oregon, USA) and Alexa Fluor 568 goat anti‐mouse antibody for 60 min at room temperature. After washing with PBS, the slides were mounted on Vectashield (Vector Laboratories, Inc., Burlingame, CA, USA) and examined using a Leica DMi8. Pictures were recorded by using 200× magnification with the same excitation setting in order to compare the different conditions.

### 2.7 Cell cycle analysis by Flow cytometry

For the cell cycle analysis, transfected cells (NC and targeting) were seeded, allowed to attach, and then treated with calcitriol. After 24 h, the cells were harvested, washed twice in PBS, and fixed in precold 70% ethanol at 4 °C overnight. Afterward, the cells were stained with propidium iodide (PI) and measured by flow cytometry (Becton, Dickinson and Company, Franklin Lakes, NJ, USA).

### 2.8 Cell target modulation analysis by western blotting

After 24 h of treatment, transfected cells (NC and targeting) were harvested and lysed on ice in cell lysis buffer (Cell Signalling Technology, Danvers, MA, USA). Total proteins were measured with the Bio‐Rad Protein Assay (Bio‐Rad Laboratories, Hercules, CA, USA). Fifty microgram of proteins were electrophoretically separated on Mini‐Protean TGX Precast Gels (Bio‐Rad Laboratories) by SDS/PAGE. The proteins were then transferred to PVDF membranes using the Trans‐Blot Turbo Mini PVDF Transfer Packs (Bio‐Rad Laboratories). The membranes were incubated with primary antibodies at 4 °C overnight and HRP‐conjugated secondary antibodies; EC Clarity Western ECL Substrate was used for antibody detection (Bio‐Rad Laboratories). Images were captured by using ChemiDoc (Bio‐Rad Laboratories).

### 2.9 Immunoassay for 17β‐estradiol determination

For 17β‐estradiol quantification, SUM149PT cells were treated for 48 h with 100 nm calcitriol and then harvested, washed three times in PBS 1×, and then lysed and sonicated. After clarification, total proteins of the lysate were measured by Bio‐Rad Protein Assay (Bio‐Rad Laboratories) to allow sample normalization. One hundred microliter of cell lysate was utilized for 17β‐estradiol quantification by the eletrochemiluminescence immunoassay (ECLIA) Elecsys Estradiol III kit–Roche Canada on COBAS analyzer, according to the manufacturer's instructions. The minimum detectable dose of 17β‐estradiol was 5 pg·mL^−1^. The measurements were run in triplicates and were preceded by blank.

### 2.10 ERRα knockdown procedure

For transient siRNA transfection, the cells were transfected using the siPORT‐NeoFX Transfection Reagent (Thermo Fisher). The siPORT‐NeoFX agent was diluted at 1 : 20 in the OPTI‐MEM medium (Thermo Fisher) and mixed to the ERRα siRNA (s4830) and Silencer® Select Negative Control siRNA (4390843) to allow transfection complex formation (siRNA 5 nm); the mixture was then dispensed into six‐well plates containing the cell suspension. Transfected cells were incubated in cell culture condition ready for assay. All the cells were tested for ERRα downregulation and siRNA was considered efficient when the ERRα expression was inhibited by at least 60–70% compared with the Select Negative Control siRNA as shown in Fig. S1.

### 2.11 Immunoassay for 17β‐estradiol determination

For 17β‐estradiol quantification, SUM149PT cells were treated for 48 h with 100 nm calcitriol and then harvested, washed three times in PBS 1×, and then lysed and sonicated. After clarification, total proteins of the lysate were measured by Bio‐Rad Protein Assay (Bio‐Rad Laboratories) to allow sample normalization. One hundred microliter of cell lysate was utilized for 17β‐estradiol quantification by the eletrochemiluminescence immunoassay (ECLIA) Elecsys Estradiol III kit (Roche Diagnostics GmbH, Mannheim, Germany) on COBAS analyzer, according to the manufacturer's instructions. The minimum detectable dose of 17β‐estradiol was 5 pg·mL^−1^. The measurements were run in triplicates and were preceded by blank.

### 2.12 Interactome analysis

An extended network was built through BioGRID data using PSICQUIC (Proteomics‐Standard‐Initiative‐Common‐QUery‐InterfaCe) for the retrieval of interaction data to identify the interactors of VDR and ESRRA. VDR and ESRRA were then queried and Pathlinker was used to identify the shortest path network. All the steps were performed in cytoscape v.3.7.1 (Institute for Systems Biology, Seattle, WA, USA). Moreover, to obtain a directed network through the Cluepedia+Cluego app, the subnetwork was enriched with information derived from the STRING database.

### 2.13 Pathway cross talk analysis in TCGA BReast CAncer dataset

RNA‐Seq FPKM, survival data, and molecular subtype information were retrieved with the tcgabiolinks package (2.13.6) [30]. The starbiotrek package (1.10.0) was used to perform pathway cross talk analysis [31]. In particular, Biocarta pathway information was integrated with PHint (PHysical interaction) network data. Basal cases were dichotomized for ESRRA expression through the ‘dichotomize’ function of the binda package (1.0.3). The survival curves were obtained, and the log‐rank test was performed with the survival r package (3.1.8). Additionally, MAF files of the TCGA‐BRCA cohort have been downloaded and annotated with oncoKB‐annotator. BRCA1 alterations with clinical level of evidence >3 have been retained, and basal‐like cases carrying relevant alteration have been dichotomized according to simultaneous coexpression of VDR‐CYP24A1‐ESRRA. All the analyses were carried out in the r 3.6 environment (R Foundation, Vienna, Austria).

### 2.14 Statistical analyses

Gene expression data, namely delta–delta Ct values, were compared through an analysis of variance model (ANOVA). The fitted model was then analyzed through a *post hoc* test (Tukey Honest Significant Differences, ‘TukeyHSD’ function) to know which pairwise comparison was significant. Wilcoxon signed‐rank test was used to perform the comparison between the data of treated *vs* vehicle‐treated cells. The ‘stats’ r package was used (r v3.5) and *P*‐values were considered to be significant when *P* ≤ 0.05.

## 3 Results

### 3.1 ERRα, VDR, and RXR basal expression in tested breast cancer cells

**Fig. 1:**
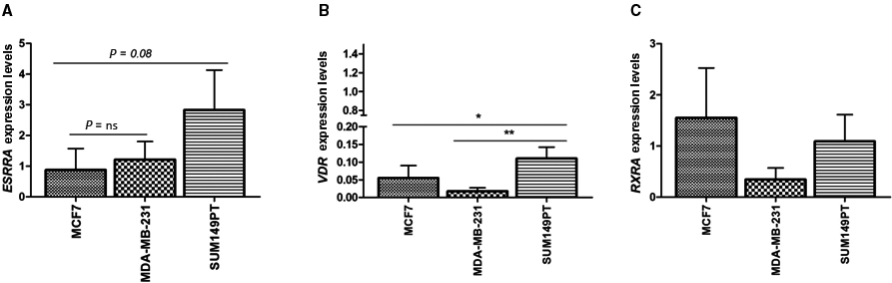
The basal levels of the three nuclear receptors transcripts are different in MCF7, MDA‐MB‐321, and SUM149PT breast cancer cells. (A) *ESSRA* gene expression, (B) VDR gene expression, and (C) RXRA gene expression were measured by qRT‐PCR. Data were normalized to the levels of RN18S1 mRNA expression and presented as 2^−ΔΔCt^. Gene expression data (ΔΔCt) were compared through an analysis of variance model (ANOVA). The fitted model was then analyzed through a *post hoc* test (Tukey Honest Significant Differences) to know which pairwise comparison was significant. Data are representative of three independent experiments performed in duplicate and represent the mean ± SD; *P ≤ 0.05; **P ≤ 0.001 *vs* MCF 10A cells, ns indicated no significance.

We first evaluated the expression levels of ERRα, VDR, and RXR transcripts in the MCF7, MDA‐MB‐231, and SUM149PT cells based on our hypothesis that these biomarkers may affect the response to calcitriol. The real‐time evaluations, performed by using bona fide normal MCF 10A cells as a reference, showed that a higher ERRα mRNA level was found in SUM149PT cells compared with MCF7 and MDA‐MB‐231 (though it did not reach the significant *P* value, *P* = 0.08); (Fig. 1A). The VDR transcript levels were lower in the MDA‐MB‐231 (*P* = 0.004) and MCF7 (*P* = 0.05) cells than in the SUM149PT (Fig. 1B) cell line; while no significant difference was found among the three cell lines in the basal RXRα mRNA levels (Fig. 1C). Since our focus was on the calcitriol degrading enzyme and estrogen signaling, we determined the basal expression levels of the CYP24A1, ERα, and CYP19A1 transcripts (Fig. S2). Collectively, these data pointed out that the SUM149PT cell line showed the highest expression levels of both the VDR and ERRα transcript, while there was no significant difference regarding the CYP24A1 and CYP19A1 expression levels. As expected, unlike MCF7, which is an ER+ luminal A breast cancer model, both MDA‐MB‐231 and SUM149PT displayed barely detectable levels of ERα since they represent triple negative breast cancer models [32].

### 3.2 Effects of the calcitriol/VDR axis: focus on the calcitriol degradation enzyme, CYP24A1, the estrogen pathway, and ERRα

**Fig. 2:**
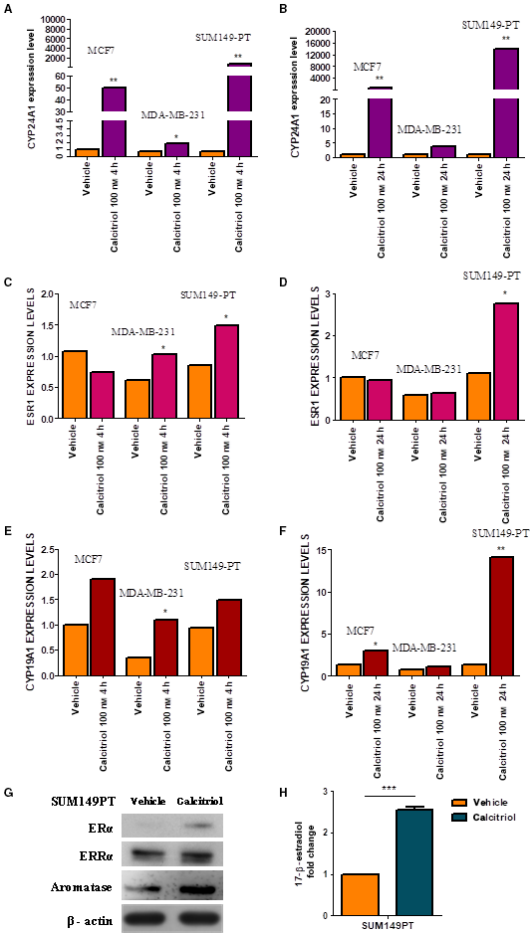
Calcitriol treatment induces the reactivation of estrogen signaling and gene involved in vitamin D metabolism in a time‐ and cell‐depend manner. *CYP24A1* fold change levels in MCF7, MDA‐MB‐321, and SUM149PT breast cancer cells by (A) 4 h and (B) by 24 h of calcitriol treatment; (C) ESR1 mRNA levels in MCF7, MDA‐MB‐321, and SUM149PT cells by 4 h of calcitriol and (D) by 24 h of calcitriol; *CYP19A1* gene expression in MCF7, MDA‐MB‐321, and SUM149PT breast cancer cells (E) by 4 h of calcitriol and (F) by 24 h of calcitriol. The gene expression experiments were performed by using the untreated cells as control, the cells treated with vehicle (DMSO), and calcitriol‐treated cells. Data were normalized to the levels of RN18S1 mRNA expression and presented as 2^−δδCt^ and analyzed by the Wilcoxon signed‐rank test. Data are representative of three independent experiments performed in duplicate; *P ≤ 0.05; **P ≤ 0.01 *vs* vehicle‐treated cells. (G) Representative images of three independent immunoblots showing the expression of ERα and aromatase in vehicle‐ and calcitriol‐treated SUM149PT. β‐actin was used as loading control. In (H) is reported 17β‐estradiol level fold change in vehicle‐ and calcitriol‐treated SUM19PT cells analyzed through paired *t*‐test. Data are representative of three independent experiments performed in triplicate and represents the mean ± SD; ***P ≤ 0.001 *vs* vehicle‐treated cells.

Next, to explore the genomic action of VDR, we challenged the cells with 100 nm calcitriol, which is the concentration generally used to study the effects of VDR activation [33]. We found that CYP24A1 transcript expression rapidly increased after 4 h of calcitriol treatment in SUM149PT cells (> 500 fold and > 50 fold over the vehicle‐treated cells; *P* = 0.004) (Fig. 2A) and further increased after 24 h of treatment (> 10 000 fold and > 1000 fold over the vehicle‐treated cells; *P* = 0.004) in MCF7 cells (Fig. 2B). CYP24A1 transcript expression increased to a lesser extent in MDA‐MB‐231 cells than in the SUM149PT and MCF7 cell lines. It was about twofold greater than in the vehicle‐treated cells (*P* = 0.01) after 4 h of calcitriol (Fig. 2A), and up to threefold greater than in the vehicle‐treated cells (*P* = 0.22) after 24 h of treatment (Fig. 2B). Given that Santos‐Martinez *et al*. [17] reported that 100 nm calcitriol induced the expression of a functional ERα in the MDA‐MB‐231 cell line, and we hypothesized a functional interaction between VDR and ERRα that may activate estrogen signaling, we also determined the effect of calcitriol on the expression of ESR1 and CYP19A1 transcripts. We found that 100 nm calcitriol induced a time‐dependent stimulation of ESR1. ESR1 transcript levels were more than onefold higher in SUM149PT cells than in the vehicle‐treated cells (*P* = 0.03) by 4 h, and more than threefold higher than in the vehicle‐treated cells (*P* = 0.02) (>) (Fig. 2C) after 24 h of calcitriol treatment (>) (Fig. 2D), while a transient stimulation of ESR1 transcript occurred only after 4 h (*P* = 0.01) in MDA‐MB‐231 cells (Fig. 2C). Calcitriol did not significantly modulate ESR1 gene expression in MCF7 cells (Fig. 2C,D). A slight but significant induction of CYP19A1 transcription (*P* = 0.03) occurred by 4 h (Fig. 2E) in the MDA‐MB‐231 cells, but it was no longer detectable after 24 h of treatment (Fig. 2F). CYP19A1 transcript levels increased in the MCF7 cells (> 1 fold higher than in the vehicle‐treated cells *P* = 0.04) and to a much greater extent in the SUM149PT cells by 24 h (> 14 fold higher than in the vehicle‐treated cells *P* = 0.007) (Fig. 2F). To further assess the reactivation of estrogen signaling, we also determined the expression of ERα and aromatase at protein level in SUM149PT cell line upon calcitriol treatment. Additionally, we determined the effect of calcitriol on ERRα protein expression in order to assess whether it upregulated the ERRα‐dependent signaling pathway. According to transcripts expression in SUM149PT cells, we found that calcitriol restored the expression of ERα and caused the increase in aromatase at protein level. Of note, in contrast to transcript expression, calcitriol determined the increase in ERRα at protein level in SUM149PT, thus suggesting that the treatment augmented the protein stability in such cells. The immunoblots are reported in Fig. 2G. Regarding CYP19A1, to demonstrate enzyme functionality we carried out experiments to quantify the synthesis of one of the main estrogens, 17β‐estradiol, upon calcitriol treatment. We found that 17β‐estradiol was barely detectable in SUM149PT cells whereas 48 h of calcitriol treatment induced 2.5 fold increment respect to baseline level (Fig. 2H). Collectively, our findings demonstrated that in the SUM149PT cell line calcitriol strongly induced the expression of its degrading enzyme (CYP24A1) as well as of key estrogen signaling biomarkers. We thus chose the SUM149PT cell line to assess the role of ERRα in the biological behavior of VDR in a representative model of triple negative, inflammatory breast cancer falling within the most aggressive basal‐like subtype of BC (BLBC), and the MCF7 cell line for the same purpose in a Luminal A breast cancer model that is less invasive and aggressive.

### 3.3 ERRα loss of function abrogated VDR‐mediated transcription on CYP24A1, ERα, and CYP19A1, but activated that on KDM1A

**Fig. 3:**
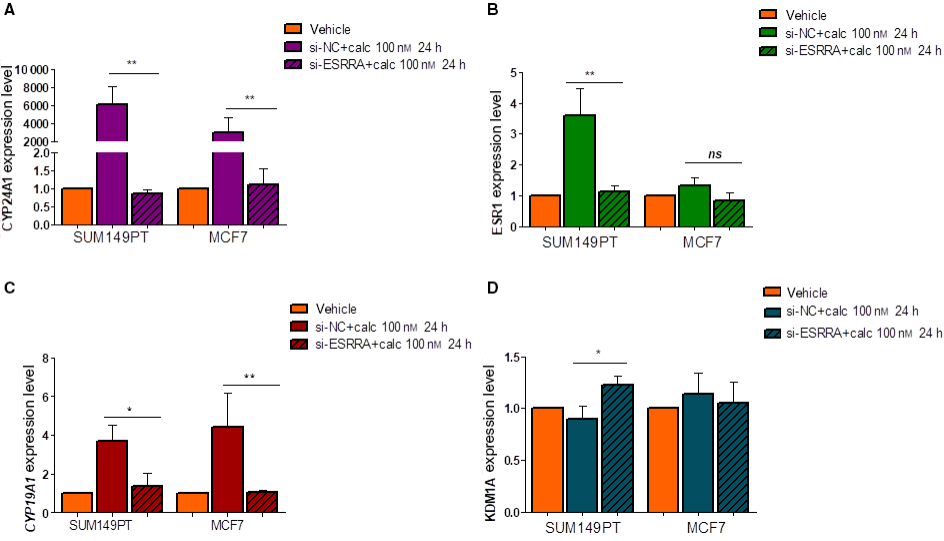
Effects of ERRα knockdown on VDR genomic action in MCF7 and SUM149PT breast cancer cells. Analysis for (A) CYP24A1, (B) ESR1, (C) CYP19A1, and (E) KDM1A mRNA in MCF7 and SUM149PT cells undergoing calcitriol treatment and si‐ERRα. Moreover, (D) the impact of ERRα knockdown on KDM1 transcript was showed. Data were normalized to the levels of RN18S1 mRNA expression and presented as 2^−ΔΔCt^. Gene expression data (ΔΔCt) were compared through an analysis of variance model (ANOVA). The fitted model was then analyzed through a *post hoc* test (Tukey Honest Significant Differences) to know which pairwise comparison was significant. Data are representative of three independent experiments performed in duplicate and represents the mean ± SD; *P ≤ 0.05; **P ≤ 0.01 *vs* vehicle cells and calcitriol empty vector cells or only empty vector cells.

To investigate the biological function of ERRα on calcitriol/VDR genomic action, MCF7 and SUM149PT cell lines were treated with 100 nm calcitriol, after the cells had been transfected with siRNA‐targeting ERRα or with NC. Knockdown of ERRα restored the basal expression of CYP24A1 in both SUM149PT (*P* = 0.0003) and MCF7 (*P* = 0.01), thus completely abrogating the effect of calcitriol on its degrading enzyme (Fig. 3A). Remarkably, ERα expression also decreased and returned to its basal level in SUM149PT cells (*P* = 0.0008) (Fig. 3B), and the same happened to CYP19A1 transcript in both MCF7 (*P* = 0.009) and SUM149PT cell lines (*P* = 0.03) (Fig. 3C). These results suggest that ERRα was a crucial regulator for VDR to initiate a genetic program leading to calcitriol degradation and activation of estrogen signaling. Of note is that this phenomenon occurred to a higher extent in the basal‐like model than in the luminal A model. Recently, Battaglia *et al*. [26] reported that LSD1 mediated the epigenetic corruption of vitamin D signaling in prostate cancer, and Carnesecchi *et al*. [27, 28] reported a close interaction between ERRα and LSD1 to regulate each other, mostly in cancer cell invasive behavior. In particular, the authors showed that LSD1 was involved in the maintenance of ERRα protein stability, while the ERRα protein induced LSD1 to erase repressive marks *in vitro*, thereby promoting the transcriptional activation of genes involved in the invasion of the extracellular matrix. Hence, we explored the effect of ERRα silencing on KDM1A expression upon calcitriol treatment to gain insights into the functional interaction of ERRα and KDM1A in VDR signaling in BC. Interestingly, ERRα silencing did not alter KDM1A expression in either cell line (Fig. S3) while calcitriol treatment significantly upregulated the mRNA expression of KDM1A only in transfected SUM149PT cells (Fig. 3D).

### 3.4 Effect of ERRα knockdown on cell clonality, calcitriol cytotoxicity, and underlying mechanisms

**Fig. 4:**
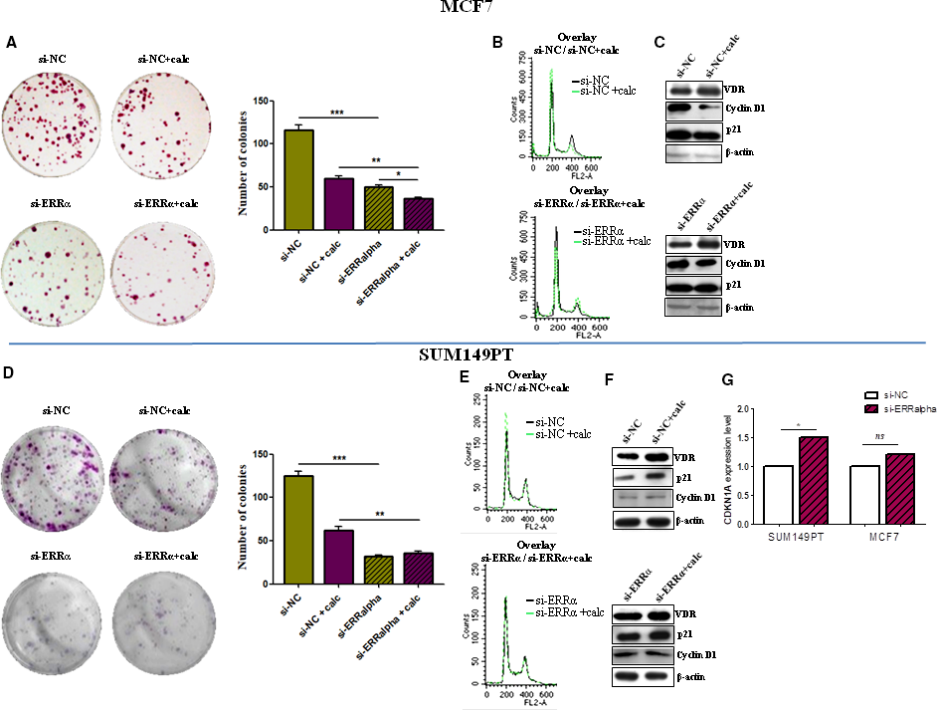
ERRα supports cells proliferation and serves calcitriol antitumor action. (A) Left, clonogenic survival assays in MCF7 cells treated with calcitriol IC_50_ concentration or/and 5 nm NC/si‐ERRα. Right, statistics of the number of colonies is shown. Data are representative of three independent experiments performed in duplicate and represent the mean ± SD. Data were analyzed by paired *t*‐test; *P ≤ 0.05, **P ≤ 0.01, ***P ≤ 0.001 calculated *vs* si‐NC. (B) The overlay of the cell cycle analysis in MCF7 cells treated with 100 nm calcitriol or/and 5 nm NC/si‐ERRα, assessed by FCM. Data are representative of three independent experiments performed in duplicate. (C) Representative images of three independent immunoblots showing the expression of cyclin D1 and p21 after treatment with 100 nm calcitriol or/and 5 nm NC/si‐ERRα in MCF7 cell line. β‐actin was used as loading control. (D) Left, clonogenic survival assays in SUM149PT cells treated with calcitriol IC_50_ concentration or/and 5 nm NC/si‐ERRα. Right, statistics of the number of colonies is shown. Data are representative of three independent experiments performed in duplicate and represent the mean ± SD. Data were analyzed by paired *t*‐test; *P ≤ 0.05, **P ≤ 0.01, ***P ≤ 0.001 calculated *vs* si‐NC. (E) The overlay of the cell cycle analysis in SUM149PT cells treated with 100 nm calcitriol or/and 5 nm NC/si‐ERRα, assessed by FCM. Data are representative of three independent experiments performed in duplicate. (F) Representative images of three independent immunoblots showing the expression of cyclin D1 and p21 after treatment with 100 nm calcitriol or/and 5 nm NC/si‐ERRα in SUM149PT cell line. β‐actin was used as loading control. (G) CDKN1A mRNA levels in MCF7 and SUM149PT cells undergoing ERRα silencing. Data are representative of three independent experiments performed in duplicate and represent the median value. *P* ≤ 0.05 *vs* NC‐ERRα. Data were normalized to the levels of RN18S1 mRNA expression and presented as 2^−ΔΔCt^ and analyzed by the Wilcoxon signed‐rank test; *P ≤ 0.05, ns indicated no significance.

To assess whether ERRα influenced tumor cell proliferation and sensitivity to calcitriol, we first tested the effect of single treatments either calcitriol or ERRα knockdown on cell clonality and then we tested the effect of the combined treatment. The results of colony formation assays indicated that (a) calcitriol induced a concentration‐dependent reduction in the numbers and size of colonies in both cell lines, with MCF7 cells being the most sensitive to calcitriol (data reported as supplementary material, Fig. S4), (b) ERRα knockdown significantly reduced colony formation in both cell lines (Fig. 4A–D), and (c) by contrast, ERRα silencing abrogated calcitriol cytotoxicity in SUM149PT cells and strongly reduced it in the MCF7 cell line. Calcitriol reduced colony formation in MCF7 much less than in nonsilenced cells (si‐NC + calcitriol) (Fig. 4A–D). Since estrogens preferentially induce cyclin D1 to trigger breast cancer proliferation while p21 is transcriptionally regulated by ERRα to remove constraints in tumor progression [34], we evaluated the function of ERRα in the expression of these targets and in VDR protein expression to explore the potential regulatory mechanism of sensitivity to calcitriol mediated by ERRα. We found that calcitriol induced an increase in VDR protein expression in both cell lines in ERRα‐silenced cells and in ERRα‐expressing cells (transfected with si‐NC), meaning that VDR activation occurred [35] irrespective of ERRα expression. However, by comparison, calcitriol reduced cyclin D1 expression in si‐NC‐MCF7 (control) cells to a much greater extent than in si‐ERRα‐MCF7 cells, while no effect was observed on p21 expression in both. By contrast, calcitriol increased p21 expression in si‐NC‐SUM149PT cells much more than in si‐ERRα‐SUM149PT cells, while no variation was found for cyclin D1 expression (Fig. 4C–F). Accordingly, the data on gene expression showed that p21 was regulated by ERRα in SUM149PT cells, as ERRα silencing significantly upregulated p21 in the SUM149PT cell line and not in MCF7 cells (Fig. 4G). Target modulation was reflected at the level of cell cycle progression. Calcitriol caused G0/G1 phase cell cycle arrest in both SUM149PT and MCF7 cells while combination with ERRα‐targeting treatment abrogated the effect of calcitriol on the cell cycle in both cell lines (Fig. 4B–E). Collectively, the results indicated that ERRα supported proliferation in both cancer models. Our findings suggested that, although a preferential involvement of ERRα conveyed sensitivity to calcitriol in SUM149PT cells while ERα did so in MCF7 cells, ERRα was crucial for the tumor‐suppressive ability of calcitriol in both tumor models, which is in line with the ability of ERRα and ERα to interfere and collaborate each other as demonstrated by their coregulation of several common target genes [36].

### 3.5 VDR and ERRα cellular localization in MCF7 and SUM149PT cells after calcitriol treatment

**Fig. 5:**
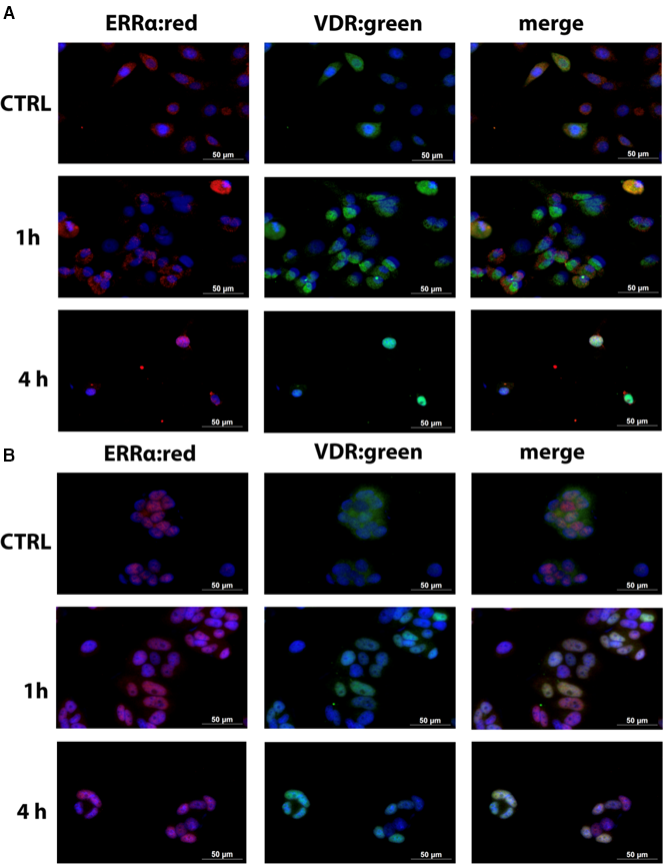
Immunofluorescence images showing the colocalization of ERRα and VDR. Representative images of (A) MCF7 cells and (B) SUM149PT cells after 1 and 4 h of calcitriol treatment. Data are representative of three independent experiments performed in duplicate. Scale bar = 50 µm.

To further address a VDR and ERRα interaction, we performed immunofluorescence analysis to visualize the cellular distribution of VDR and ERRα in calcitriol‐treated cells *vs* vehicle‐treated cells. As shown in Fig. 5A, time‐dependent nuclear accumulation of VDR and ERRα was observed in SUM149PT cells, in which both nuclear receptors basically colocalized after treatment with calcitriol. The MCF7 cell line showed a higher basal ERRα expression in the nucleus, unlike VDR. Upon calcitriol treatment both ERRα and VDR increased in the nucleus (Fig. 5B). Consistent with data we reported before, immunofluorescence results suggest that VDR and ERRα interact and that their interaction is ligand‐dependent in SUM149PT cells and ligand‐enhanced in MCF7 cells. To examine whether this was a result of a direct interaction, we performed a bioinformatic analysis.

### 3.6 Interactome analysis of VDR/ESRRA axis

**Fig. 6:**
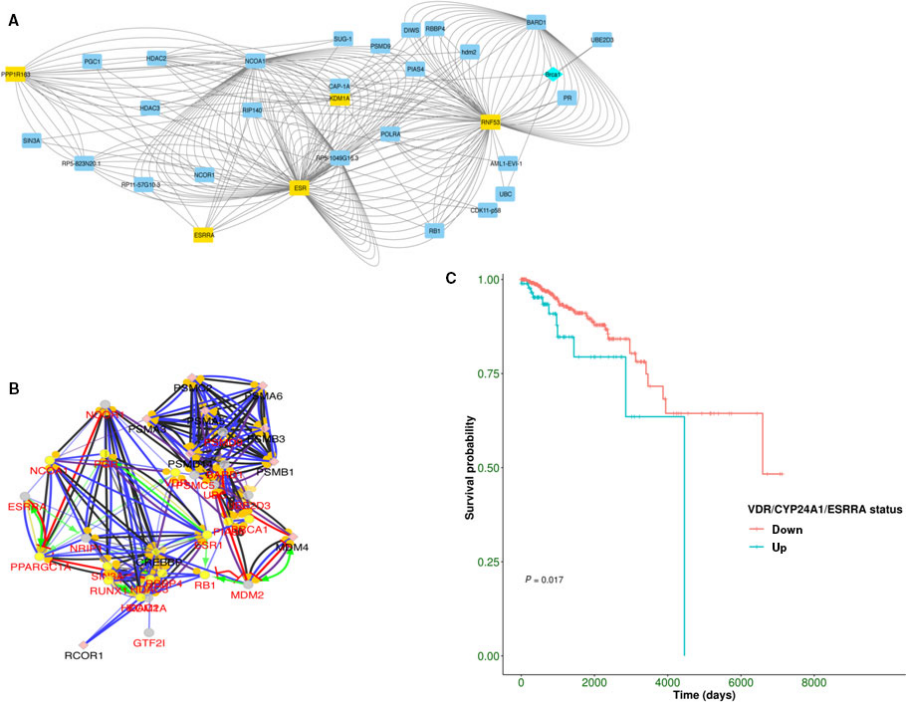
Interactome analysis and survival in the TCGA dataset. (A) BioGRID network including known interactors of VDR and ERRα. Blue rectangles are human genes, yellow rectangles indicate main protein hubs, instead light blue rhombus indicates the mouse Brca1 gene; (B). STRING‐based network enriched with the types of interactions linking nodes; (C) Kaplan–Meier curves and log‐rank test comparing overall survival of TCGA cases with simultaneous overexpression of VDR‐CYP24A1‐ERRα (blue curve) and those without (red curve).

An interactome analysis was set up. Through BioGRID, we built an extended network to query VDR(PPP1R163), ERRα (ESRRA), ERα (ESR), BRCA1(RNF53), and KDM1A as main interacting protein hubs in human. Such a choice was based on ERRα‐interacting proteins emerged by our study and by recent report showing a direct interaction between ERRα and BRCA1 in BRCA1‐mutated carriers [37], which is a setting represented by the SUM149PT cell line in our experiments, while others have demonstrated a direct interaction between ERRα and KDM1A [27, 28]. The subnetwork, identified from the whole database (Fig. 6A), evidenced a cluster of 31 interacting proteins in which VDR, ERRα, ERα, BRCA1, and KDM1A emerged as main interacting protein hubs in human (highlighted in yellow rectangles in Fig. 6A). The analysis showed also Brca1 in light blue because it is the mouse protein which has been expressed in a human cell line, thus resulting in mouse–human interactions. Such subnetwork was further analyzed through the STRING interaction database. This further analysis allowed to better explore the BioGRID subnetwork, highlighting the specific types of interactions connecting the nodes (Fig. 6B). Of note, PGC‐1α played a central role in the directed network, connecting VDR and ESRRA. Because of the latter result, we also investigated the effect of calcitriol on PGC‐1α transcript expression in both MCF7 and SUM149PT to assess its involvement in VDR/ESRRA axis. The results of such analysis (Fig. S5) showed that calcitriol induced an increase in PGC‐1α transcript, though it was not significant respect to the baseline expression level in both cell lines.

### 3.7 From biological network to pathway cross talk

The impact of our *in vitro* results was studied in the TCGA‐BRCA cohort. Cases were selected according to molecular subtypes in order to reflect the setting of cell lines, namely Basal‐like (SUM149PT) and Luminal A (MCF7). After the selection step, the *in silico* cohort included 567 patients with Luminal A BC and 194 patients with basal‐like BC.

In order to assess the pathway activity related to the VDR/ERRα axis, the FPKM values of the KDM1A, BRCA1, and PPARGC1A genes were also included in the cross talk analysis, given the roles they proved to have in our *in vitro* experiments and according to our interactome results. The StarBioTrek package was used because it is more informative than enrichment analysis in providing information on pathways and their relative cross talk integrating networks and gene expression data. We found ‘control of gene expression by vitamin d receptor’/‘regulation of pgc‐1a’ and ‘pelp1 modulation of estrogen receptor activity’/‘control of gene expression by vitamin d receptor’ cross talks with AUC values of 0.55 and 0.52, respectively, using Biocarta pathway data integrated with PHint network information. We thus tried to identify the biological role of ESRRA by dichotomizing the basal‐like subset for its expression. Interestingly, we detected the same cross talks as in the previous comparison with AUC values of 0.67 and 0.66, respectively, in the ESRRA overexpressing group. Such a result is promising because ESRRA stratification is able to biologically discriminate basal cases with more elevated AUC values than a basal‐like *vs* Luminal A group analysis.

Additionally, we evaluated the expression of PGC‐1α in the basal‐like *in silico* cohort stratified according to ESRRA expression. Such analysis evidenced that PGC‐1α is upregulated in the ESRRA overexpressing patients (mean = 0.665 ± 1.95), while it is downregulated in the ESRRA downexpressing patients (mean = 0.243 ± 0.48). The difference was statistically significant (*P* = 0.004).

### 3.8 Translational significance of ERRα/VDR axis and survival in TCGA dataset

Literature data and our *in vitro* and *in silico* results left the prognostic value of the VDR‐CYP24A1‐ERRα axis open to question. Overall survival data of basal‐like patients were downloaded and the patients were stratified into two groups according to whether VDR‐CYP24A1‐ERRα simultaneous overexpression was present or not. The Kaplan–Meier curves (Fig. 6C) and log‐rank test showed that patients overexpressing VDR‐CYP24A1‐ERRα genes had a significantly worse survival than the other group (*P* value = 0.017), clearly indicating a prognostic value of such a biomarker signature for basal‐like breast cancer.

Additionally, by speculating on the role of BRCA1 mutation on ERRα/VDR axis in basal‐like tumors, we performed the analysis by queering how are BRCA1‐mutated patients divided over the VDR‐CYP24A1‐ERRα downexpressing and highexpressing signature group. The results showed that only ten over twenty basal‐like were BRCA1‐mutated patients in the TCGA‐BRCA cohort (data reported in Table S1) and all ten showed downregulation of VDR‐CYP24A1‐ERRα signature.

## 4 Discussion

To the best of our knowledge, no evidence has been reported on the interplay between VDR signaling and ERRα in breast cancer. In this study, by hypothesizing a convergence of signaling, we uncovered a novel ERRα/VDR axis through which ERRα promoted a putative mechanism of vitamin D deficiency and deregulation of VDR genomic action by activating estrogen signaling in breast cancer cell lines. Here, ERRα was identified as a calcitriol‐induced regulator of both VDR genomic action and VDR antitumor action in either ER‐positive and ER‐negative breast cancer models. Functionally, ERRα sustained the proliferation of BC cell lines and upregulated the expression of CYP24A1 (the enzyme that catalyzes calcitriol degradation), ERα, and CYP19A1 in calcitriol‐treated cells. The re‐expression of functional ERα in triple negative breast cancer cell lines upon calcitriol treatment has already been reported [17]. Here, we showed that calcitriol caused both the re‐expression of ERα and the increase in a functional aromatase, further reinforcing the link already showed between ERRα/ERα signaling in BC [37] and lending credence to the notion that ERRα altered the VDR effect on estrogens. ERRα silencing functionally reduced the calcitriol‐dependent inhibition of clonogenic survival. Although the latter appears to be a controversial result, it may be explained whether we hypothesize potential points of ERRα‐ERα cross talk. There is growing evidence that calcitriol promotes breast cancer‐protective actions in ERα‐positive tumors, mostly because it constrains estrogen signaling effects [4]. We found that calcitriol reduced the clonogenic survival of both MCF7 and SUM149PT cells, while inducing ERα expression in the SUM149PT cell line. Therefore, we can speculate that an ERα‐dependent activity of ERRα mediated the antiproliferative function of calcitriol in both cell lines. Since estrogens preferentially induce cyclin D1 to trigger breast cancer proliferation while p21 is transciptionally regulated by ERRα to remove constraints on tumor progression [34], we evaluated the effect of ERRα on the expression of such targets. Through loss of function experiments, we demonstrated that ERRα abrogated calcitriol‐induced upregulation of p21 in SUM149PT cells and strongly reduced calcitriol‐induced downregulation of cyclin D1 in MCF7 cells. Such target modulation was also reflected in cell cycle progression and clonogenic survival, further supporting the notion that ERRα‐ERα cross talk regulated sensitivity to calcitriol in both cancer models, while ERRα caused deregulation of VDR genomic action mostly in the basal‐like model. After a well‐known ERRα regulator, KDM1A, [30] was recently observed to be involved in the corruption of vitamin D signaling in prostate cancer, we assessed the ERRα‐KDM1A connection in the VDR pathway of BC. We found that KDM1A expression was upregulated by silencing ERRα in calcitriol‐treated SUM149PT cells, basically suggesting that the ERRα‐containing complex represses KDM1A transcription when VDR is activated by calcitriol. We found that calcitriol increased the ERRα protein expression in SUM149PT and since KDM1A is also involved in maintaining ERRα protein stability [27], we can speculate that KDM1A upregulation by calcitriol may have compensated the loss of ERRα, by sustaining ERRα expression to promote ERRα‐dependent deregulation of the VDR pathway. The bioinformatics analysis we carried out provided evidence of an interacting network in the ERRα/VDR axis and although interactions retrieved from BioGRID repository were referred to different cell types, this strengthened our hypothesis regarding the connections between ERRα and ERα and between ERRα and KDM1A. Of note, in line with the pivotal role of PGC‐1α as a key regulator of metabolic reprogramming in advanced cancer [38, 39], PGC‐1α emerged as a central mediator in the directed network connecting VDR and ESRRA, thus supporting the notion that a PGC‐1α /ERRα‐containing complex drives a program that alters vitamin D metabolism in advanced breast cancer. Furthermore, since a high ERRα expression has been associated with tumor aggressiveness [19], we performed a pathway cross talk analysis that measured the activity of pathways and their relationships to provide evidence of the biological effects triggered by ERRα overexpression. The analysis showed a cross talk between ‘control of gene expression by VDR’ and the ‘regulation pathway of PGC‐1α’, and in addition, we found that PGC‐1α was upregulated in ERRα overexpressing basal‐like cohort, strengthening the hypothesis on the existence of an interaction between the VDR/ERRα axis and PGC‐1α‐dependent metabolic function. A connection was also detected between ‘control of gene expression by VDR’ and the ‘PELP1 modulation of estrogen receptor activity’, indicating cross talk between the VDR/ERRα and PELP1/ERα pathways in patients.

**Fig. 7:**
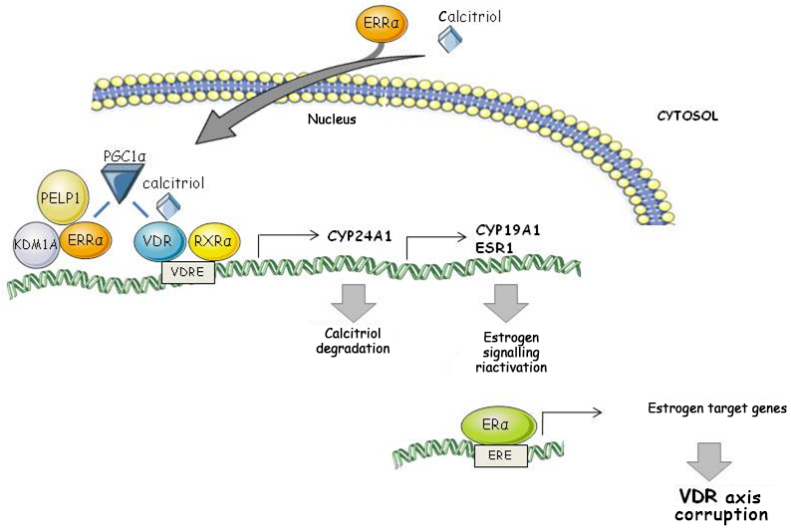
Graphical representation of the complex ERRα/PGC‐1α/VDR mediating gene expression regulation in loci in which ERRα acts as regulator of VDR. Calcitriol promotes the translocation of ERRα from cytosol to nucleus. We thus hypothesize a model of mediated gene regulation in which PGC‐1α plays a key role by coupling VDR with ERRα. The latter NR (nuclear receptor) acts as either regulator of VDR and as connecting protein between VDR and estrogen signaling, by interacting with PELP1 and KDM1A. This transcriptional complex ERRα/PGC‐1α/VDR boosts the expression of CYP24A1 and induces the expression of ERα and CYP19A1.

PGC‐1α is a coactivator of VDR [40] and a regulator of ERRα [19, 41], while PELP1 is a coactivator of ERα and it is involved in epigenetic modifications of the aromatase promoter through interactions with ERRα and KDM1A [42, 43] to induce *in situ* estrogen synthesis. We thus hypothesized a model of ERRα/PGC‐1α/VDR‐mediated gene regulation in which ERRα acts as a VDR regulator and as the protein connecting VDR and estrogen signaling to induce estrogen activation, perhaps by modulating the demethylating activity of KDM1A through interaction with PELP1 (Fig. 7). Since the best record in terms of pathways cross talk was achieved in the BLBC setting and, collectively, our findings supported the view that (a) ERRα deregulated VDR function mostly when it was highly expressed in the BLBC setting and (b) calcitriol induced an increase in VDR and CYP24A1 expression in both *in vitro* models, we assessed the prognostic significance of a simultaneous overexpression of ERRα, VDR, and the target gene CYP24A1 in both BLBC and in BRCA1‐mutated subgroup to gain insights on possible role that BRCA1 status might have on ERRα/VDR axis. This approach pointed out the translational potential of such a signature, by showing that overexpression of all three biomarkers definitely defined a poor prognosis in BLBC patients and may be correlated with a reduction in circulating calcitriol. Of note, although we found only ten BRCA1‐mutated patients within BLBC, all showed the simultaneous downregulation of VDR‐CYP24A1 and ERRα, suggesting that BRCA1 status might be correlated to potentially different biological effect of ERRα/VDR axis, which warrants further investigations.

## 5 Conclusions

Our findings pointed out that (a) ERRα plays a role in vitamin D metabolism and sensitivity in breast cancer, (b) the ERRα/VDR axis is at the crossroads of estrogen signaling activation, and (c) the simultaneous overexpression of ERRα, VDR, and CYP24A1 is correlated with poor prognosis in basal‐like breast cancer.

Collectively, our results confirm ERRα as a master regulator of oncometabolic and proliferating signals in breast cancer, and provide insights into the molecular mechanisms underpinning VDR genomic and antitumor action in advanced breast cancer. ERRα may lead to a defective vitamin D pathway, which, as suggested by Feldman *et al*. [4], would make vitamin D administration less effective or even harmful in this setting.

## Conflict of interest

The authors declare no conflict of interest.

## Author contributions

KD and LP designed, performed the experiments, and wrote the manuscript; SDS performed all statistical and bioinformatic analyses; RDF and SS performed western blot and immunofluorescence experiments and assisted in figure preparation; BP and RL editing of the manuscript; AA and SS supervised research, reviewed the manuscript, and involved in funding acquisition. All authors read and approved the final manuscript.

## Supporting information


**Fig. S1.** All transfected cells were tested for the downregulation of ESSRA. Silencing of ESRRA was considered efficient when the expression of the nuclear receptor was inhibited by at least 60%‐70% compared with select negative control siRNA (si‐NC). Transcript levels were measured by qRT‐PCR. Data were normalized to the levels of RN18S1 mRNA expression and presented as 2^‐δδCt^. Gene expression data (δδCT) were compared through an analysis of variance model (ANOVA). The fitted model was then analyzed through a *post hoc* test (Tukey Honest Significant Differences) to know which pairwise comparison was significant. Data are representative of three independent experiments performed in duplicate and represent the mean ± SD; **: *P* ≤ 0.01, ***: *P* < 0.001.

**Fig. S2.** The basal levels of **a** *CYP24A1*, **b** *ESR1*, and **c** *CYP19A1* genes are showed in MCF7, MDA‐MB‐321, and SUM149PT breast cancer cells. Transcript levels were measured by qRT‐PCR. Data were normalized to the levels of RN18S1 mRNA expression and presented as 2^‐δδCt^. Gene expression data (δδCT) were compared through an analysis of variance model (ANOVA). The fitted model was then analyzed through a *post hoc* test (Tukey Honest Significant Differences) to know which pairwise comparison was significant. Data are representative of three independent experiments performed in duplicate and represents the mean ± SD; *: *P* ≤ 0.05 *vs* MCF 10A cells.

**Fig. S3.** Effects of ERRα knockdown on *KDM1A* gene expression. Transcript levels were measured by qRT‐PCR. Data were normalized to the levels of RN18S1 mRNA expression and presented as 2^‐δδCt^. Gene expression data (δδCT) were compared through an analysis of variance model (ANOVA). The fitted model was then analyzed through a *post hoc* test (Tukey Honest Significant Differences) to know which pairwise comparison was significant. Data are representative of three independent experiments performed in duplicate; ns: not significant.

**Fig. S4.** Representative images of clonogenic survival assay performed in **a** SUM149PT cells and **b** MCF7 cells treated with different concentration of calcitriol. n = 3 independent experiments in duplicate were performed. **c** Dose response plots showing clonogenic survival percentage calculated versus vehicle‐treated cells. The concentration yielding 50% inhibition of clonogenic survival (IC_50_) was calculated by Calcusyn software.

**Fig. S5.** Calcitriol induced the increase in PGC‐1α transcript expression level in both MCF7 and SUM149PT cell lines respect to vehicle‐treated cells, though it was not significant. The gene expression experiments were performed by using the untreated cells as control, the cells treated with vehicle (DMSO), and calcitriol‐treated cells. Data were normalized to the levels of RN18S1 mRNA expression and presented as 2^‐δδCt^. Data, analyzed by Wilcoxon signed‐rank test, are median value of three independent experiments performed in duplicate; ns: not significant.

**Table S1.** Table reporting the list of the TCGA‐BRCA cohort of patients carrying deleterious BRCA1 alteration and the relative molecular subtype.

## Data Availability

The datasets analyzed during the current study were generated by the TCGA Research Network (https://www.cancer.gov/tcga) and are available in the GDC data portal repository (https://portal.gdc.cancer.gov/projects/TCGA‐BRCA).
