# Supplementary figures and images for: The ERRα–VDR axis promotes calcitriol degradation and estrogen signaling in breast cancer cells, while VDR‐CYP24A1‐ERRα overexpression correlates with poor prognosis in patients with basal‐like breast cancer
Source: Mol Oncol. 2021 Jul 16;16(4):904–20. doi: 10.1002/1878-0261.13013 (PMC8847991; doi:10.1002/1878-0261.13013)

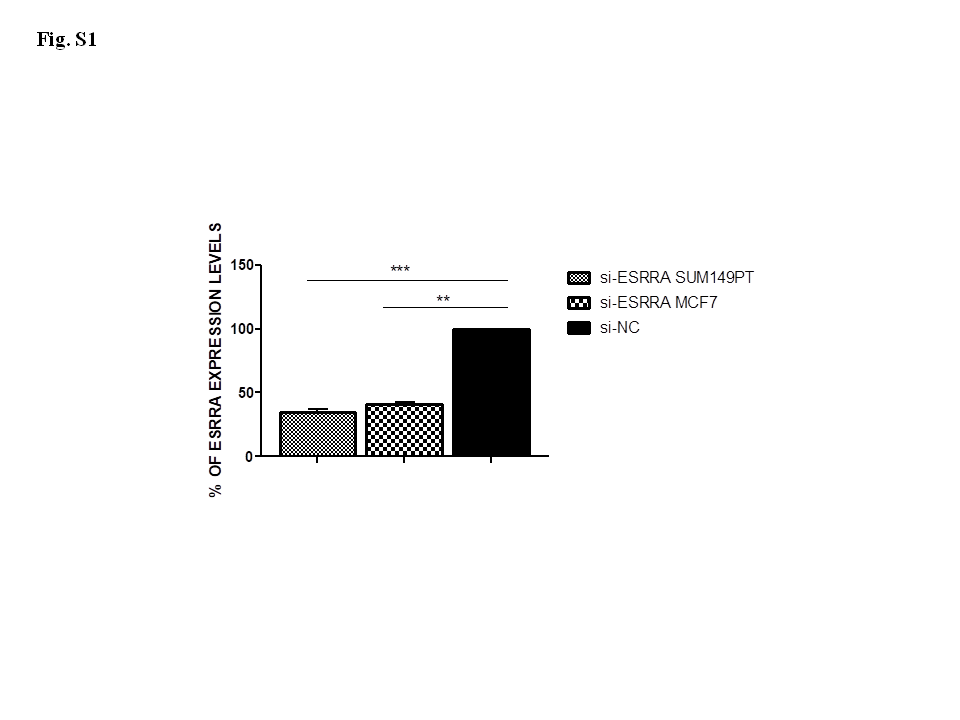

Supplement: Supplementary file 1 — Fig. S1. All transfected cells were tested for the downregulation of ESSRA. Silencing of ESRRA was considered efficient when the expression of the nuclear receptor was inhibited by at least 60%‐70% compared with select negative control siRNA (si‐NC). Transcript levels were measured by qRT‐PCR. Data were normalized to the levels of RN18S1 mRNA expression and presented as 2‐δδCt. Gene expression data (δδCT) were compared through an analysis of variance model (ANOVA). The fitted model was then analyzed through a post hoc test (Tukey Honest Significant Differences) to know which pairwise comparison was significant. Data are representative of three independent experiments performed in duplicate and represent the mean ± SD; **: P ≤ 0.01, ***: P < 0.001. [file MOL2-16-904-s005.tif]

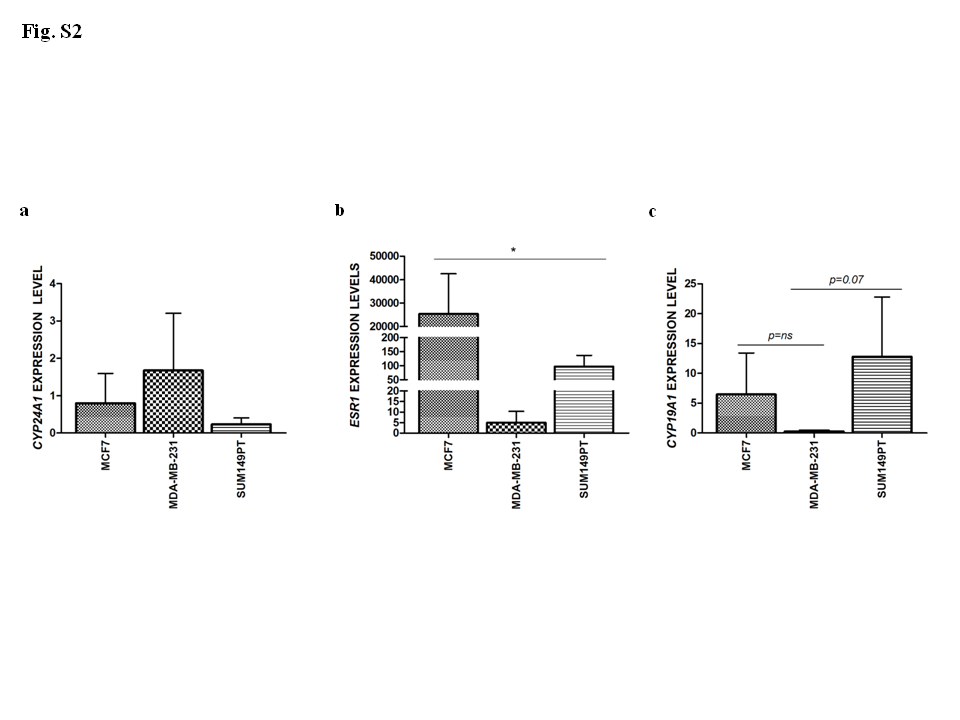

Supplement: Supplementary file 2 — Fig. S2. The basal levels of a CYP24A1, b ESR1, and c CYP19A1 genes are showed in MCF7, MDA‐MB‐321, and SUM149PT breast cancer cells. Transcript levels were measured by qRT‐PCR. Data were normalized to the levels of RN18S1 mRNA expression and presented as 2‐δδCt. Gene expression data (δδCT) were compared through an analysis of variance model (ANOVA). The fitted model was then analyzed through a post hoc test (Tukey Honest Significant Differences) to know which pairwise comparison was significant. Data are representative of three independent experiments performed in duplicate and represents the mean ± SD; *: P ≤ 0.05 vs MCF 10A cells. [file MOL2-16-904-s002.tif]

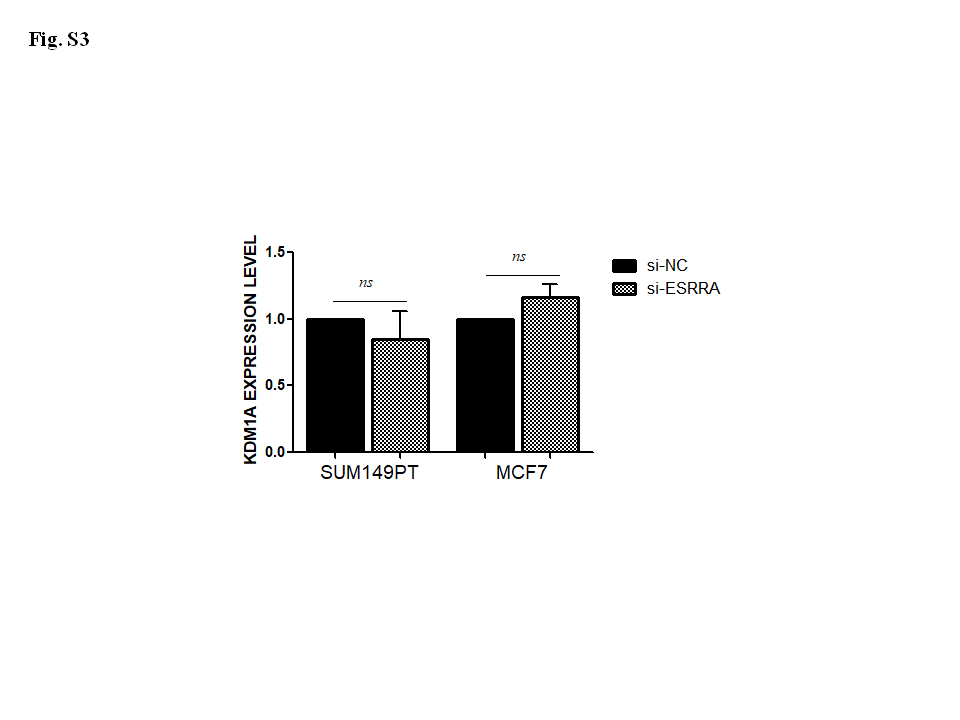

Supplement: Supplementary file 3 — Fig. S3. Effects of ERRα knockdown on KDM1A gene expression. Transcript levels were measured by qRT‐PCR. Data were normalized to the levels of RN18S1 mRNA expression and presented as 2‐δδCt. Gene expression data (δδCT) were compared through an analysis of variance model (ANOVA). The fitted model was then analyzed through a post hoc test (Tukey Honest Significant Differences) to know which pairwise comparison was significant. Data are representative of three independent experiments performed in duplicate; ns: not significant. [file MOL2-16-904-s003.tif]

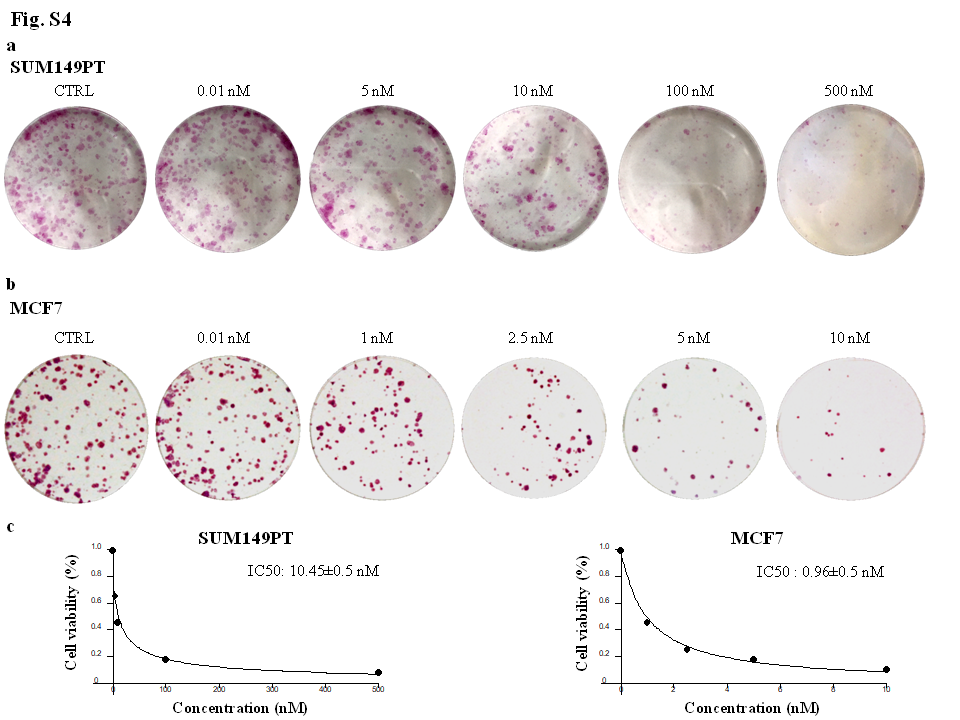

Supplement: Supplementary file 4 — Fig. S4. Representative images of clonogenic survival assay performed in a SUM149PT cells and b MCF7 cells treated with different concentration of calcitriol. n = 3 independent experiments in duplicate were performed. c Dose response plots showing clonogenic survival percentage calculated versus vehicle‐treated cells. The concentration yielding 50% inhibition of clonogenic survival (IC50) was calculated by Calcusyn software. [file MOL2-16-904-s006.tif]

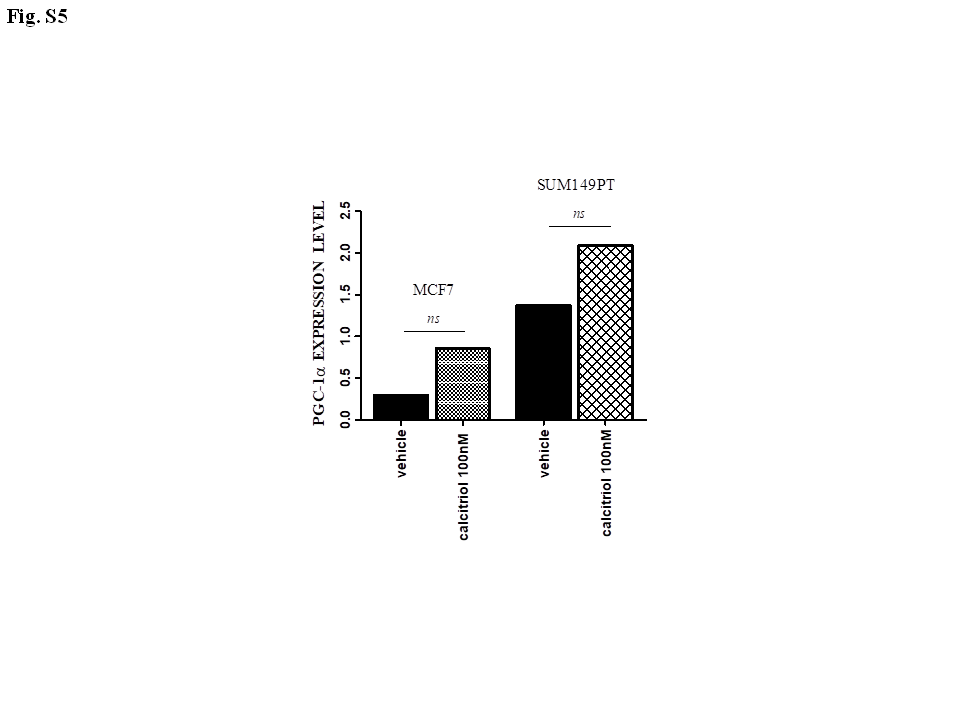

Supplement: Supplementary file 5 — Fig. S5. Calcitriol induced the increase in PGC‐1α transcript expression level in both MCF7 and SUM149PT cell lines respect to vehicle‐treated cells, though it was not significant. The gene expression experiments were performed by using the untreated cells as control, the cells treated with vehicle (DMSO), and calcitriol‐treated cells. Data were normalized to the levels of RN18S1 mRNA expression and presented as 2‐δδCt. Data, analyzed by Wilcoxon signed‐rank test, are median value of three independent experiments performed in duplicate; ns: not significant. [file MOL2-16-904-s001.tif]
