# Supplementary material for: The ERRα–VDR axis promotes calcitriol degradation and estrogen signaling in breast cancer cells, while VDR‐CYP24A1‐ERRα overexpression correlates with poor prognosis in patients with basal‐like breast cancer
Source: Mol Oncol. 2021 Jul 16;16(4):904–20. doi: 10.1002/1878-0261.13013 (PMC8847991; doi:10.1002/1878-0261.13013)
Supplement: Supplementary file 6 — Table S1. Table reporting the list of the TCGA‐BRCA cohort of patients carrying deleterious BRCA1 alteration and the relative molecular subtype. [file MOL2-16-904-s004.pptx]

## Slide 1
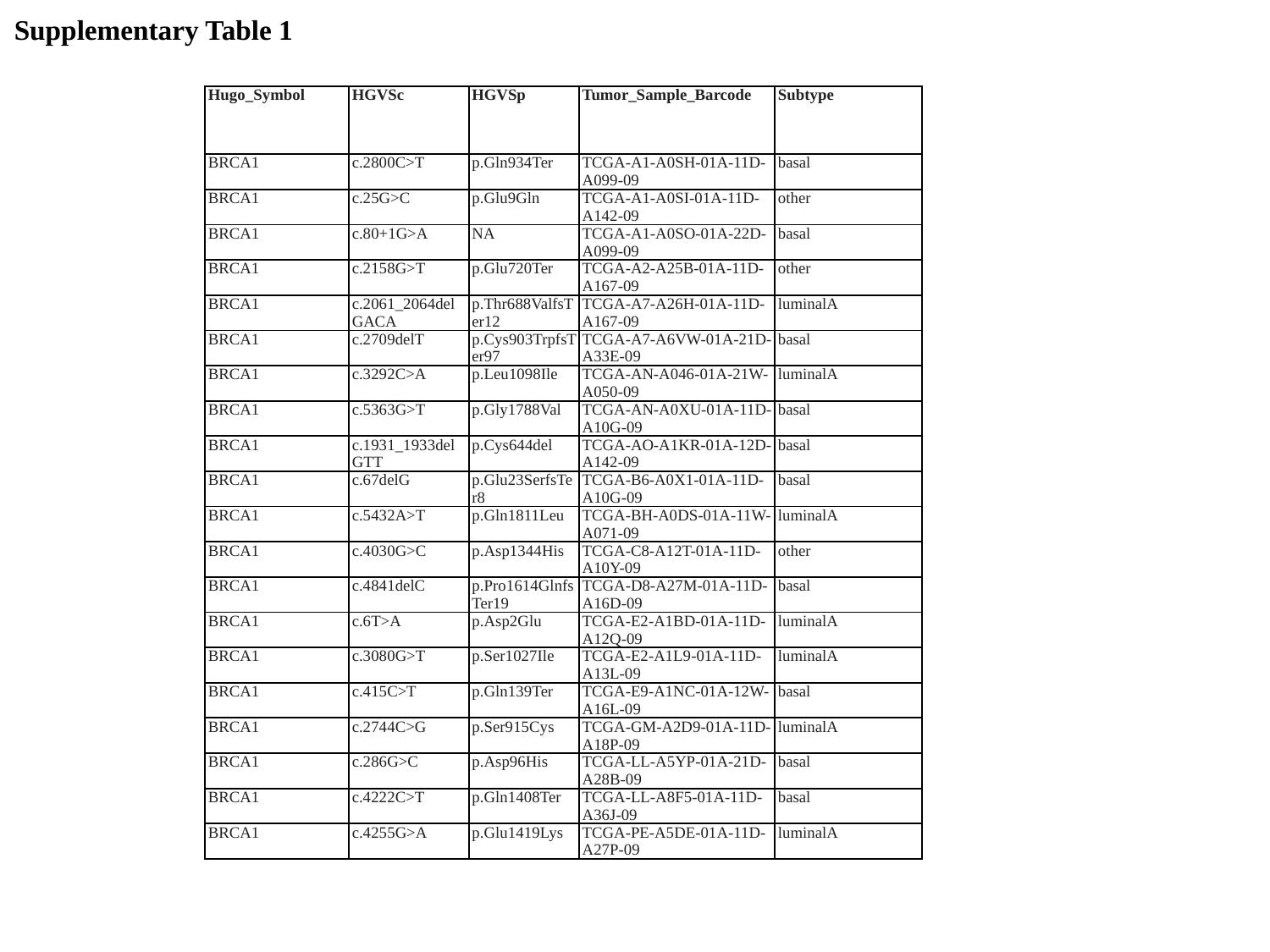

Supplementary Table 1
| Hugo\_Symbol | HGVSc | HGVSp | Tumor\_Sample\_Barcode | Subtype |
| --- | --- | --- | --- | --- |
| BRCA1 | c.2800C>T | p.Gln934Ter | TCGA-A1-A0SH-01A-11D-A099-09 | basal |
| BRCA1 | c.25G>C | p.Glu9Gln | TCGA-A1-A0SI-01A-11D-A142-09 | other |
| BRCA1 | c.80+1G>A | NA | TCGA-A1-A0SO-01A-22D-A099-09 | basal |
| BRCA1 | c.2158G>T | p.Glu720Ter | TCGA-A2-A25B-01A-11D-A167-09 | other |
| BRCA1 | c.2061\_2064delGACA | p.Thr688ValfsTer12 | TCGA-A7-A26H-01A-11D-A167-09 | luminalA |
| BRCA1 | c.2709delT | p.Cys903TrpfsTer97 | TCGA-A7-A6VW-01A-21D-A33E-09 | basal |
| BRCA1 | c.3292C>A | p.Leu1098Ile | TCGA-AN-A046-01A-21W-A050-09 | luminalA |
| BRCA1 | c.5363G>T | p.Gly1788Val | TCGA-AN-A0XU-01A-11D-A10G-09 | basal |
| BRCA1 | c.1931\_1933delGTT | p.Cys644del | TCGA-AO-A1KR-01A-12D-A142-09 | basal |
| BRCA1 | c.67delG | p.Glu23SerfsTer8 | TCGA-B6-A0X1-01A-11D-A10G-09 | basal |
| BRCA1 | c.5432A>T | p.Gln1811Leu | TCGA-BH-A0DS-01A-11W-A071-09 | luminalA |
| BRCA1 | c.4030G>C | p.Asp1344His | TCGA-C8-A12T-01A-11D-A10Y-09 | other |
| BRCA1 | c.4841delC | p.Pro1614GlnfsTer19 | TCGA-D8-A27M-01A-11D-A16D-09 | basal |
| BRCA1 | c.6T>A | p.Asp2Glu | TCGA-E2-A1BD-01A-11D-A12Q-09 | luminalA |
| BRCA1 | c.3080G>T | p.Ser1027Ile | TCGA-E2-A1L9-01A-11D-A13L-09 | luminalA |
| BRCA1 | c.415C>T | p.Gln139Ter | TCGA-E9-A1NC-01A-12W-A16L-09 | basal |
| BRCA1 | c.2744C>G | p.Ser915Cys | TCGA-GM-A2D9-01A-11D-A18P-09 | luminalA |
| BRCA1 | c.286G>C | p.Asp96His | TCGA-LL-A5YP-01A-21D-A28B-09 | basal |
| BRCA1 | c.4222C>T | p.Gln1408Ter | TCGA-LL-A8F5-01A-11D-A36J-09 | basal |
| BRCA1 | c.4255G>A | p.Glu1419Lys | TCGA-PE-A5DE-01A-11D-A27P-09 | luminalA |
